# Domination and resistance in school – The Alternative School Day for ‘at-risk students’: What does it mean for them to be there, and to what extent do they benefit?

**DOI:** 10.1080/02673843.2014.948019

**Published:** 2014-12-08

**Authors:** Lillian Lundstrøm, Lisbet Øygard

**Affiliations:** ^a^ Department of Psychosocial Science, UIB, Christiesgt.13, 5015Bergen, Norway

**Keywords:** evaluation of alternative school day (ASD), better approval in ASD, the regular school as a middle-class sphere, both domination and resistance in regular school, resistance as a mechanism of keeping one's self-esteem

## Abstract

Alternative School Day (ASD) is a project for adolescents who have difficulties in several areas. Nine pupils (14–16-years-old), their parents and teachers were interviewed during autumn and spring. The pupils attended ASD one day per week. All of them had a working-class background. The study focuses on the school as a middle-class arena, and for pupils with other class background, it represents an ‘away ground’. Bourdieu's concepts of cultural capital and habitus, and Giroux's emphasis of individuals as intentional actors, have been used to study domination and resistance in schools. The findings may indicate that there exist both domination and resistance in schools.

## Introduction

The Norwegian school has been, and still is, strongly influenced by the illusions of equality and democracy. The school should be a good place for learning and personal growth for all. However, studies have shown that there exists a discrepancy between ideology and praxis (Ogden, 1998). Not all pupils feel that they fit into school. It has been argued that schools reproduce differences that already exist in the society, and in some cases, the gap increases (Nordahl, 2010). Status within the school is connected to an understanding that good grades offer opportunities in the educational system and, later on, in the labour market. These days, grades are considerably more important for what the future might bring than they were previously. Therefore, adolescents now-a-days must struggle to a high degree for a position in society. The conditions were considerably different 40–50 years ago, because one could finish school after seven years and begin to work. It has been debated how ideal it was to start working so early since many wanted to consider their education, but were unable to for financial reasons. The problem today is that there exist normative demands that one should at least complete college, and many from the working class and the lower middle class fulfil college and also get university degrees. Social mobility through ‘class journeys’ through education has therefore increased and given the youth new possibilities (Solvang, 2002). At the same time, pupils who do not feel they fit into school will become even worse off since many consider school to be meaningless.

Pupils who do not fit into school are exposed to different forms of adjustment pressure. And pupils with externalizing behavior may be evaluated as candidates for special treatments. Previously, we had B-classes for pupils who were not as ‘clever’ or ‘adjusted’ as those in the A-classes, and there existed more special schools. Today, many of these special initiatives have been implemented into the ordinary schools and the initiatives are more directed at the particular pupil (NOU, 2003). In Norway, this is supported by The Norwegian Learning Plan from 1997 (KUF, 1996). This may, for instance, include extra teacher or assistant resources. In 1987, it was decided as a principal rule that pupils with problems should get education within the framework of regular classes. Inclusion was formulated as a value in itself and grounded in the principle of democratic participation (Arnesen, 2002, p. 55). Heggen, Jørgensen, and Paulsgaard (2003, pp. 106–107) claim that schools do little to deal with the problems of the marginalised pupils by finding ways of solving the difficulties. They argue that school resources monopolised by ordinary classroom education adversely affect pupils who are in risk zones.

In this article, a school initiative, the ‘Alternative School Day’ (ASD), will be studied. The Church City Mission is responsible for the project. ASD is a preventive arrangement for adolescents in risk zones, that is, they have difficulties in several arenas, such as school, at home or in their spare time. Pupils who are between 14 and 16 years of age participate, and the schools choose the pupils who get the opportunity to participate, but the pupils and their parents have to give their permission. The pupils are taken out of regular school to attend ASD for one day per week throughout the school year, that is, they begin in August and finish in June. Significant targets are to promote the adolescents' possibilities for learning, coping and personal responsibility. Individual strategies are developed for each pupil in cooperation with schools on the basis of individual educational plans. By identifying the pupils' life situation, plans are worked out for how one can work best with them. It is regarded as important that the pupils obtain reliable and positive contact with the staff at ASD. Consequently, there are two adults in the school groups and a maximum of six pupils.

## Theoretical contributions to explain domination and resistance in schools

Bourdieu and Passeron (1977) have developed a sociology of curriculum that links culture, class and domination, on the one hand, and schooling, knowledge and biography, on the other. They argue that schools institutionalise, through the rules and meanings that constitute the day-to-day working of classroom experience, the dominant cultural capital. Schools play a crucial role in reproducing the unequal distribution of cultural capital. Instead of providing compensatory education to the students with different cultural capital, the school, while appearing neutral, asks them to think and perform in a way that is quite alien to their own background. The concepts of cultural capital and habitus are central to understand Bourdieu's analysis of how the mechanisms of cultural reproduction function concretely within schools. Cultural capital refers to different sets of linguistic and cultural competencies that individuals inherit by way of the class-located boundaries of their families. A child inherits from his/her family a set of meanings, qualities of style, modes of thinking, and types of dispositions that are accorded a certain social value and status as a result of what the dominant class or classes label as the most valued cultural capital. Schools play a particularly important role in both legitimating and reproducing the dominant culture, for example, students whose families have a tenuous connection to forms of cultural capital highly valued by the dominant society are at a decided disadvantage. Class and power connect with dominant cultural production not only in the structure and evaluation of the school curriculum, but also in the dispositions of the oppressed themselves, who actively participate in their own subjugation. This becomes clearer if we examine Bourdieu's notion of *habitus.* According to Bourdieu, habitus refers to the subjective dispositions which reflect a class-based social grammar of tastes, knowledge and behaviour inscribed permanently in the ‘body schema and the schemes of thought’ of each developing person. Habitus, or internalised competencies and sets of structured needs, represents the mediating link between structures, social practice and reproduction. That is the system of ‘symbolic violence’ does not mechanically impose itself on the oppressed; it is at least in part reproduced by them, because the habitus governs practices that assign limits to its ‘operations of invention’. In other words, objective structures – language, schools and families – tend to produce dispositions, which in turn structure social experiences that reproduce the same objective structures.

Giroux (2001, pp. 107–111) agrees with Bourdieu regarding the significance of cultural capital and habitus in schools. However, he has criticised Bourdieu's theoretical advances for being over-determined. He argues: ‘The notion of habitus is in linking the concept of domination to the structure of personality needs, its definition and use constitute a conceptional straitjacket that provides no room for modification to escape’ (Giroux, 2001, pp. 90–91). Furthermore, Giroux argues that Bourdieu disregards the assumption that reflexive thought may result in social practices that qualitatively restructure one's dispositions or structure of needs, one's habitus.

Giroux maintains that the oppressed are not viewed as being simply passive in the face of domination. The notion of resistance points to the need to understand more thoroughly the complex ways in which people mediate and respond to the interface between their own lived experiences and structures of domination and constraint. Central categories that emerge in the problematic of resistance are intentionality, consciousness, the meaning of common sense, and the nature and value of non-discursive behaviour. Furthermore, resistance adds new theoretical depth to Foucault's (1977) notion that power works so as to be exercised on and by people within different contexts that structure interacting relations of dominance and autonomy. In the behaviour of subordinate groups, there are moments of cultural and creative expressions that are informed by a different logic, whether it be existential, religious or otherwise. Giroux claims that resistance has to be situated in a perspective or rationality that takes the notion of emancipation as its guiding interest. Resistance needs to be viewed from a very different theoretical starting point, one that links the display of behaviour to the interests it embodies. Moreover, he argues that there is a need to reformulate the relationship among ideology, culture and hegemony to make clear the ways in which these categories can enhance the understanding of resistance as well as how such concepts can form the theoretical basis for a radical pedagogy that takes human agency seriously.

In the present study, we have investigated the understanding of the pupils' school situation by asking the pupils, their parents and the teachers. The main issue was to study the pupils' experiences in schools in order to examine domination and resistance in this institution. Secondly, we wanted to study whether these groups perceive the situation differently or whether there is a connection between the understandings.

## Methods

### The qualitative research interview

A qualitative interview is defined as an interview whose purpose is to obtain descriptions of the life world with respect to interpreting the meaning of the described phenomena (Kvale, 1996, p. 21). The interview form treated here is a ‘semi-structured’ interview: It has a sequence of themes to be covered, as well as suggested questions. At the same time, there is an openness to changes of sequence and forms of questions in order to follow up the answers given and the stories told by the subjects. There are no methods to arrive at essential meanings and deeper implications of what has been said in an interview. In this study, meaning condensing was used; this means an abridgment of the meanings expressed by the interviewees into shorter formulations. For example, several teachers formulated ‘he doesn't understand anything regarding theoretical subjects, but he's clever in gymnastics and mechanical subjects’. This statement was condensed to ‘he's weak in theoretical subjects, but copes well in practical subjects’.

### Sample, interview guides and reliability

Eight boys and one girl, their parents and teachers were interviewed twice – in the autumn (T1) and during the spring (T2). The main focus at both evaluations was the pupils' school situation. At T1, all the pupils were interviewed face-to-face in a locale at ASD. At T2, five were interviewed at the same place, one at his home and three by telephone, because they travelled away on their summer holiday. The teachers received the questions via email before they were interviewed. Because a tape recorder may disturb the respondents, it was decided to do the note taking on the spot.

The interviews were transcribed and the written text was the material for the subsequent interpretation of meaning. Transcribing the interviews from an oral to a written mode structures the interview conversations in a form amenable to closer analysis. Structuring the material into text facilitates an overview that is in itself a beginning analysis (Strauss & Corbin, 1990, p. 30).

The pupils were split into three groups divided over three days. As mentioned, there was one girl in the sample. This represented a problem when providing them with pseudonyms to protect their identity. The solution was that all the pupils acquired a boy's name. All the pupils had a working-class background.

For both T1 and T2, three interview guides were constructed. These contain questions to the pupils, their parents and teachers. The first interview with the pupils covered areas such as the school situation, relationships with parents and friends, and their spare time. At T2, it was focused on possible changes since T1, for example, whether they shirked school (played truant) more or less, whether their relationships with parents and teachers were changed, and whether they themselves had changed in some way. Questions to parents at T1 involved whether they thought that their children would benefit from participating at ASD, and conditions regarding their children's school and family situation. The questions to teachers at T1 involved what kind of influence they thought ASD would have upon the pupils' social and professional management, level of truancy, and whether they had been involved in bullying, conflicts with teachers and other pupils. At T2, it was primarily focused on possible changes since T1 for both parents and teachers.

There was a strong correspondence between what the pupils and their parents told. However, the teachers often had a different view. Nevertheless, it is possible that the questions directed to the pupils were not always adequate because it was relatively common that the pupils answered ‘I don't know’ or ‘I have not thought about this’.

### The adolescents at ASD

The pupils were divided into three tentative groups: The marginalised (M-group), the twilight zone (T-group) and the integrated (I-group). This is a way to operationalize the concept of behavior delinquencies, and to clear the material. Some of the pupils could be referred to as marginalised, but were situated under the twilight zone because they were better in some arenas. The integrated pupils were easier to place because they functioned in a more proper way than the others did. However, the three groups have two things in common. First, with one exception, they functioned well together with other pupils, and second, they did not cope with the theoretical subjects.

### The marginalised

Joachim, Jonas and Tom had a negative development during the school year. Joachim and Jonas had left school and Tom had moved to a special school. Tom resided with both parents while Joachim and Jonas lived with their mothers. All three had a bad relationship with their parents. Heggen et al. (2003) found that the marginalised adolescents compensated for the bad relationships with their parents by developing friend networks. This was not the case for Tom and Joachim because they seemed to have problems establishing close relationships with both friends and adults. Furthermore, they were depressed and had no initiative. The distanced relationship between Tom and his parents may have initiated his excitement in drug milieus, and this made the relationship even worse. Jonas was also in contact with drug milieus, but he had friends outside this milieu as well. He was in conflict with his mother, but they had contact and he often brought friends to his home. Tom chose another strategy; he was not at home in order to avoid quarrelling and fuss. Both Tom and Jonas had been in trouble with the police several times; Tom for selling and using drugs, and Jonas for stealing. These boys had withdrawn from family and school, which can be seen as an attempt to avoid social control in arenas that have great value for adolescents' integration in society.

### The twilight zone

Lars and Adrian had left school – Lars had started to work, and Adrian had moved to a special school. Even though they were not at ordinary school any longer, they were better off than the M-group. Both Lars and Adrian reported that they had a close and good relationship with their parents. Lars lived with both his parents while Adrian resided with his father. It was common that the pupils had most contact with their mothers so the bond between Adrian and his father represented the exception. Lars played football and this means a lot to him, although he had few friends in this milieu.

### The integrated

Andy, Sam, Martin and Tony were still at the ordinary school at the end of the school year. All four had a positive development from T1 to T2. They seldom shirked school, but did sometimes show up late. Furthermore, all were engaged in sport activities, and they had friends in these milieus. They had a relatively good relationship with their parents, although they sometimes quarrelled with them. In addition, Andy was the only one who did not reside with both parents. Finally, they had better relationships with the teachers than the others.

### ASD as a comfort zone, relationships with the adults and identity development

Experienced quality of life is to a great extent related to a subjective feeling of comfort or absence of this. Furthermore, to feel comfortable includes: to feel at home, to be seen, to be taken seriously and that what one does is experienced as meaningful (Berger, 2000). It is obvious that the adolescents felt they were at home at ASD, and that this represented a substantial contrast to how they experience school. All pupils report that they felt comfortable at ASD. Tony reported that ‘*We are like a big, happy family’.* Furthermore, Joachim's teachers said that he shirked school and had great problems with getting out of bed in the mornings, but that he always showed up at ASD, and that ASD offered him a day with positive experiences. Tom's father was also very positive, and he told that his son was very satisfied at ASD. It was very pleasant for him to see that Tom was satisfied because he had great problems at school.

However, ASD was not only a comfort zone. The project also gave the pupils a feeling of freedom, that is, freedom from school, as well as a place where they could mend their ways. Joachim said that it had meant a lot for him to be here because it was a break from school, but also because the adults treated him respectfully, and he respected them. This was in great contrast to his relationships with the teachers. Martin said that it was easier talking to the staff at ASD than the teachers because they understood him much better. Furthermore, Andy reported that at ASD he felt that he was an important person. He also felt that he was very clever, and that was very different from what he felt at school.

It is obvious that the pupils' satisfaction at ASD was related to a break from school and to do something they experienced as meaningful. They participated in activities they otherwise did not have access to.

A central factor related to the pupils' contentment was the significant contact that they achieved with the adults. All of them reported that they listened to them, helped them, and that they were met with respect and understanding. ASD may therefore have contributed to a noteworthy contact with adults. Jonas' teacher told that it was important for him to be acquainted with adults because the teachers did not have capacity to take care of all the pupils. She also told that she did not manage to get a close contact with him, but she believed that the adults at ASD had. Adrian's teacher also said that she did not have the capacity to take care of all the pupils, and she thought it was very important for Adrian to get support and confirmation from the adults at ASD. She also said that ASD had given him a better self-esteem and that he had become happier and more secure. Furthermore, Martin's teacher told that ASD had given Martin self-confidence because he had got many practical challenges that he coped with to a much higher degree than the theoretical subjects at school.

A positive identity development occurred among most of the pupils. The majority reported that they felt more self-secure, and this was probably because they got new coping experiences and approval from the adults. Copings can therefore constitute a break with the ‘loser role’. ASD has through its praxis helped the pupils to look upon themselves in more positive terms. This concerns primarily the I-group, but the T-group also showed a constructive growth as a result of the participation at ASD. On the contrary, the development was negative through the school year for the M-group. Tom became more depressed and used more drugs while Joachim isolated himself more than before. However, Tom, Joachim and Jonas reported that ASD was important for them. They always showed up, they felt comfortable there and got positive feedback on themselves. One may therefore conclude that the adults at ASD have made considerable progress with the pupils. The staff did therefore serve as supervisors, encouragers and as trustworthy professionals for both the pupils and their parents.

### The school situation

The school situation represented an extensive problem for the adolescents. At T2, only four of them were still at ordinary school. Of the others, two attended special schools, two had not been at school for the last two months before the end of the school year, and one had begun working. Heggen et al. (2003, p. 57) claim that the pupil role defines a major status because school brings them together in a predictable way. It is a stable source regarding establishment and maintenance of friendships and social networks. In this perspective, the majority of the pupils were badly positioned because five of them were not at regular school any longer. Nevertheless, it is important to note that Tom and Adrian reported that they were much more satisfied after they moved to special schools. They achieved a good relationship with their new teachers, they got more attention and stopped shirking school. Tom's father told that his son had learned more in the six months he had been at special school than in the rest of his school years.

### The social life

The adolescents reported that they had satisfactory relationships with their classmates. None of them were involved in bullying, but previously some of them had bullied others or others had bullied them. As mentioned, none of them did cope with the theoretical subjects. In the study ‘Young in Norway’ from 1992, adolescents were asked to give points to different groups of adolescents regarding how much they liked/disliked them. The answers are thought-provoking. Most popular were adolescents who worked hard to get good grades (Øia, 1996, p. 25). Also pupils who did not manage the school demands had these as their ideals. Another study demonstrated that clever pupils were most popular and had a higher status in the class than the weaker ones (Arnesen, 2002, p. 319). On the basis of these findings, one may suggest that adolescents at ASD were not very popular and that few of the other pupils looked upon them as their ideals. However, the following statements illustrate that some of them were popular:

He has two friends at school and some of the others think he's cool because he makes up things. However, he doesn't have any positive contact with the other pupils. (Adrian's teacher, T1)

He talks a lot to his mates. They follow up and enjoy it. The pupils get ‘show’, otherwise the day is boring. He tells the others that he uses drugs and they think it's very exciting to hear about the drug milieu. The classroom situation is quite different when he's not at school. (Tom's teacher, T1)

He doesn't do what he's told to do. If he's informed to do something, he gets aggressive or leaves school. He doesn't want to talk to the teachers. I nearly see him; he has resigned. He wants to be cool and plays that role. He has a ‘Do-as-I-like- attitude’, and he only meets at school when it suits him. (Jonas' teacher, T2)

Some of the other pupils think he's cool because he doesn't pay any attention to the education. He's very seldom at school; he has resigned totally. (Joachim's teacher, T1)

It has been observed that pupils can try to hide their incompetence by not doing their tasks, making fuzz in order to keep their dignity (Sørlie & Nordahl, 1998). As demonstrated here, it was the M-group and Adrian from the T-group who were regarded as cool by their classmates. Tom and Adrian were popular because they made up things in such a way that the lessons were disordered. Disturbing in the *form of fuzz* can in such a perspective be seen as coping strategies as this may give them social approval from classmates. It has been observed that this especially concerns pupils who are less successful than others (Sørlie & Nordahl, 1998, p. 231). Joachim did not make any trouble; he had resigned and wanted to be left alone. His teacher told that even though he did not have anything to contribute, the others always wanted to work with him. Furthermore, these pupils were regarded as cool because they paid no attention to the teaching. The M-group and Adrian had a relatively high status in the classes because they did not follow the rules. All of them had resigned; they did not participate and did not want to have any contact with the teachers.

The teachers, especially one of them, treat us differently. The clever ones are never criticized, but I have never been treated well. (Joachim, T1)

I feel comfortable with my classmates, but the teachers are very annoying. They don't treat us equally; I'm scolded when the guilty ones don't. I think it's because they are much cleverer than I am. If I'm in trouble with the teachers, I can't do anything because they don't care anyway. (Martin, T1)

These statements illustrate that some pupils feel that they are unequally treated because they are not clever, and that they think the teacher favours those who are skilful.

The teachers don't treat me respectfully. I'm dissatisfied with everything at school, especially the teachers. I'm continually in conflict with them. (Tom, T1)

I've a bad relationship to all the teachers. They don't like me, and I don't like them. (Adrian, T1)

He nags about nothing, and criticizes the teachers, but he has good relationships to his mates. But when we are alone, he's very kind. (Tom's teacher, T1)

If he's forced to perform well at school, he become challenging, i.e. I get answers that I don't like, and we start to quarrel. Sometimes he is impudent*;* I think this is his method to demonstrate his dissatisfaction with school. (Tony's teacher, T1)

He doesn't handle the subjects. He doesn't understand the teaching, and he gets aggressive when he's forced to do something. Very often he disturbs the lessons; the classroom situation is very different when he's not present. (Adrians' teacher, T1)

I like very much being at school now. I'm not involved in any conflicts with the teachers after I attended a special school. The teachers listen to me and they respect me. I don't shirk school anymore. (Adrian, T2)

He shows up at school very seldom. He has personal problems, and he doesn't cope with school. He's very passive; he doesn't participate at all. (Lars' teacher, T1)

Previously, when he didn't understand the teaching, he interfered with the teaching. But now, he doesn't make any fuzz anymore. If he doesn't understand the lessons, he resigns. (Andy's teacher, T2)

The teachers don't have any time for me, and they don't understand me. I don't fit into school at all. (Lars, T1)

Tony and Tom demonstrated provoking behaviours. Previously Andy had too, but it seems that this has stopped and he has become passive. Lars has always been passive, but the teacher told that he was a kind pupil who never caused any troubles.

However, Tom behaved more ‘properly’ when the teacher was alone with him. This may indicate that when they were alone, he did not need to show his classmates his carelessness/neglect towards school.

Sam and Tony from the I-group also declared that they had difficulties with their teachers. However, it has been found that because of V13, they got a better connection with them.

As mentioned, Adrian attended a special school, and the statements above illustrate very well that his relationship with school and teachers has changed considerably since he was in regular school.

It was interesting to observe the discrepancy between the pupils' and the teachers' understanding of the pupils' school situation. The teachers never mentioned that there was something wrong with the school; it was the pupils who represented the problem. Whereas, the pupils reported that it was the teachers who were difficult, annoying or treated them unequally. The teachers' considerations of problem-defined behaviours were mostly related to the pupils' performances. Some of them pointed to difficult home conditions, but they never mentioned anything about school that could cause them any trouble. However, several of them were aware that they did not have capacity to take care of all the pupils, which is illustrated in some of the statements above.

### Theoretical focus and self-esteem related to school subjects

The reforms of the last years are regarded by many as a further step towards a more theoretical governed school. The new vocational subjects have got a more theoretical focus compared to the old vocational school and apprenticeship programmes. The pupils did not cope concerning theoretical subjects, but with regard to practical subjects, they managed well:

He has great difficulties in keeping up with the teaching. He's very weak. He doesn't do anything at all, but he's more active in practical subjects. Regarding theoretical subjects, he doesn't benefit from school at all. (Joachim's teacher, T1)

He's not clever at school, but he's very interested in mechanics. He's very clever with cars and has a great interest in this. (Martin's mother, T1)

I'm clever in gymnastics, otherwise I'm a failure. I can't sit quietly. Its very boring being at school. (Tom, T1)

The majority of the pupils reported that school was boring. This may reflect the fact that they do not handle the theoretical subjects. Arnesen (2002, p. 261) found that boredom appears when pupils do not understand what the teachers tell them and when they did not get enough supervision. Arnesen also found that creating a disturbance was an expression of boredom and that it was the most common way of resigning from the situation or of creating situations that could break the dullness. The school therefore appears as detached from ‘the real life’, as understood by the pupils. The feeling of doing something meaningful through school therefore becomes difficult. One may declare that school represents ‘an away ground’ for these pupils. This may indicate that many of the pupils' values, opinions, interests and activities are not valued and actively used in their education, and the pupils' subjective experiences and interests are of little importance to the teachers. This lack of valuation of adolescents' interests and values can be related to the boredom and alienation many pupils experience in school.

Lars reported that he felt like a loser because he did not understand anything of what the teachers told. His mother said that he did not function well socially because everybody knew that he was not clever. She also told that he withdrew from the others because he felt inferior.

The teachers confirmed the statements regarding theoretical weakness among the pupils. One may suggest that this has influenced their self-esteem and their self-image. Identity is created in association with others, and in fellowships patterns of expectations are created that maintain certain forms of behaviours. The person who is considered by others to be less competent has not only his/her own competence to struggle with, but also others' understandings and interpretations (Frønes, 1997, p. 46). This may contribute to the exposed person creating deviated forms of behaviours. A pattern has been formed, maybe for all the school years. A systematic non-approval may have been shaped and deepened in social interaction (Froestad & Solvang, 2000). Over time, the pupil may become associated to a social role that is unbearable for him/her, and also for others. It may create a need for escape, away from school to a milieu where one can achieve approval. Since there are only four pupils left in the ordinary school, this may have happened here. Tom and Jonas found excitement in drug milieus. Subcultures may offer a feeling of belonging that schools do not provide (Hebdige, 1979). In such a perspective, seeking out subcultures can be seen as a coping strategy towards an existing dominant culture that the school represents.

Already in 1973, Christie asks: ‘Why do not the institution change into a place where the pupils can feel comfortable. Why are not subjects thrown out and life in?’ (1973, p. 84). The pupils at ASD would profit from having more practical subjects. Several of them said that they were clever in gymnastics, art and mechanics, but because of the strong theoretical focus in schools it is not surprising that many fall by the wayside and feel that they are losers. In the end, they give up because they ‘know’ that they will not understand or cope with the demands. When they see how straightforwardly other pupils, often those from the middle class, manage the demands, this will naturally weaken their self-esteem. As a result, they may become apathetic or aggressive. Martin, Jonas, and Adrian's teachers confirmed that the pupils became aggressive because they did not manage the duties directed to them. Consequently, the alternative of further integration in ‘these preventive programmes’ becomes attractive for pupils, but also for teachers and the schools, who apparently get rid of a problem as well.

### The relationship between home and school

Some of the parents showed little interest in school; they seldom or never attended parental conferences and meetings. The reason for this is that they might feel powerless towards a *system* that has stigmatized their children as difficult and/or ‘useless’, and that is not able to value and see the resources that their children are in possession of. Mellin-Olsen and Rasmussen claim in the book ‘The School's Violence’:

The school should help and cooperate, but at the same time *the communication between school and home reproaches against the home*. The school interferes with telephones, notes and letters. In the moment when the deviance appears from the school's ideal picture, the negative communication starts flowing. (1974, p. 73)

In such a perspective, it is not surprising that some parents avoid attending school meetings even though they want their children to manage well.

There's minimal contact between family and school. His parents are skeptical towards the school. They're afraid, it's embarrassing when teachers contact them. They don't show up at conferences. It's incredible unpleasant for them to show up at school. (Martin's teacher, T1)

For the first time adults have a positive attitude towards him. The teachers have never said anything nice about him. It means a lot to me that the adults at ASD are so positive, and that they have faith in him. They say that he's kind and clever. (Jonas' mother, T2)

Ogden (1998, p. 10) maintains that the cooperation between home and school is an unused resource in the work with prevention and reduction of school-related problems. This study also demonstrated that it was the parents of children who had problems in school, who did not show up at parental conferences. In order to do something about this, Ogden argues that parents should not only be informed when there are problems, but also when the pupils behave well. Furthermore, Nordahl and Sørlie (1996, p. 126) maintain that parents, children and schools are disadvantaged by having a school that takes on a confrontational stance, and a blaming attitude towards parents who have ‘problematic’ children. An improvement of the pupils' future situation can only be achieved by a trustworthy and equivalent cooperation between the school and the home.

## General discussion

Bourdieu considers schools on the basis of domination; that is, the pupils' cultural capital and their habitus do to a great extent determine their situation and experiences in schools. Giroux recognises the importance of cultural capital, but he is also concerned with intentional behaviour, that is, pupils may act rationally and that they have different methods of resisting school. Bourdieu does not regard individual behaviours as intentional; behaviours are primarily internationalised through the individuals' biography. The findings in this study may indicate that cultural capital is of great influence regarding the pupils' school conditions. None of the pupils' parents had a higher education and some of them lived in poor conditions. In order to achieve a moderate amount of problem behaviours in school, it may be a prerequisite that pupils experience that their *values, interests and experiences are valued and used.* This is related to an understanding that pupils have their own culture or habitus, and that this habitus ought to be considered in their education (Bourdieu, 1993).

A problem regarding social integration is then that the school intentionally or unintentionally cultivates ways of behaving that are foreign for adolescents in risk zones. They have probably felt that they have been little valued by the teachers. This is in accordance with Bourdieu and Passeron's description (1977) of how teachers, often with a middle-class background, recognise the value of cultural capital that especially exists among pupils with similar background, and correspondingly react on pupils who lack this form of capital. Correspondingly, those students who lack this form of cultural capital, are not regarded as clever, and as having the “right” ways of speaking and behaving, will therefore influence the relationship with the teacher.

As mentioned, Bourdieu has been criticised for excluding the active nature of resistance in school (Giroux, 2001, p. 91). In order to explain problematic behaviour and maladjustment in school, it will be necessary to analyse the pupils' mentalities and actions within the context they are situated. Pupils who show problem behaviours and maladjustment can apparently give an expression of views of reality that are to some extent rational. Examples are that school is unimportant and not interesting, and that it is more important to have friends, than to get good grades and be educated. For some pupils this view of reality can be rational even though for them the school is not of significance. Their understandings have to be considered on the basis of the pupils' social background, experiences and actions and whether it is in accordance with teachers and schools' views of reality or not (s.61). To what extent the actions are rational can be estimated in relation to whether the chosen actions are the best means to realise an individual's wants/values on the basis of his/her views of reality (Elster, 1988, p. 30).

As we have seen, several of the pupils have resigned and have carried out different ways to resist school. In accord with Giroux, this may be seen as intentional strategies: pupils who fail may have their strategies, in order to keep their dignity, and making fuzz is one way of maintaining this.

Nevertheless, one may ask what pupils can achieve by opposing school. Previously, dissociating from school could give credit and create the basis for collective solidarity and cooperation (Willis, 1977). As Willis has demonstrated, withdrawing from the pupil role and being a part of a resistance culture is closely connected to the recognition that the investments give little credit in the form of good grades. Previously, different forms of resistance in the school protected the identity of the pupils against being defined as ‘losers’ or ‘deviants’. Nowadays, we see less of this kind of protection/resistance, or informal networks. It seems that such collectives have disappeared and replaced by individualisation. The absence of, or weak, social coping is therefore also an individual challenge. This represents a problem, both for those who do not fit in and for those who work with integration activities, such as ASD (Sørlie, 1991).

Repeated investments and efforts with minor outcomes can lead to losing a belief in ones own abilities to develop existing play strategies. Such strategies can be seen as processes of marginalisation. Giving up the pupil role, or dropping out of school, places the adolescents in a situation where they have to fill their lives with meaningful activities. In school, this may result in the form of a resistance culture. Instead of being a victim of the school's classifications, they resist the game and the game rules. Together they define their own game and game rules, grounded in social background and in opposition to teachers, the game rules in the class and the cultural capital's institutionalised forms.

Giroux (1988) claims that in a situation where the pupils' own experiences are devalued, this creates the basis for resistance in school. Something that is often expressed through oppositional behaviour, criticising, maladjustment and passivity. The resistance is intentional and involves a protection of one's own dignity. Through provoking behaviour and passivity, the pupils resist devaluation, and this can represent a way to sustain their own autonomy and subjectivity.

The results demonstrated that several of the pupils showed problematic behaviours in the form of both oppositional actions and passivity. This may interpreted as *the pupils' own experiences not are satisfactorily valued.* It is not possible to declare whether this behaviour is intentional or not. However, it is reasonable to believe that the pupils are aware that their behaviour is problematic through the negative valuation of their behaviour at school and that they are aware of what they are doing. This may also be related to the pupils' experiences of the teaching and the relationship with the teachers. On the basis of a subjectivistic position, it is therefore reasonable to claim that some of the problematic behaviours can be seen as resistance. It appears that some pupils through problematic behaviours show resistance to a school system that they are not satisfied with. The pupils show resistance in situations where they have little control and are little valued (*because their experiences, interests and norms* and *habitus are not sufficiently dealt with*). It has been argued that problematic behaviour, as resistance, appears when the pupils' cultural capital is devalued. Pupils seemingly/apparently show resistance to a conformity- and adjustment-oriented and authoritarian school that possibly does *not utilise the pupils' experiences, interests and values.* This interpretation expresses a view that does not consider pupils as passive victims for reproduction of processes in school. They may try to have an influence on their situation, through, for example, resistance and problematic acting. The low level of pupil democracy and co-determination/joint consultation may indicate that resistance is one of few possibilities the pupils have to influence their school situation (Nordahl, 1998). In this sense, there is a connection between the concept of resistance and the focus on problematic behaviours as rational actions.

A pedagogic program that is based on the teachers' own reality, that it does not take *any notice of the pupils' experience and values*, and that the pupils' task is not to disturb the teaching, may result in oppositional and externalization behaviour in form of fuzz and disturbances.

### Limitations connected to the study

In this study, we have only considered pupils from the working class, that is, it is not possible to say anything about how pupils from the middle class experience school. In addition, the individuals under investigation have problems in several areas, that is, they are not representative of the working-class pupils. Furthermore, many pupils from the middle class may experience school to be boring too, and that they may have problems in different areas as well.

## Conclusions

ASD obviously had a positive effect for the pupils in this study. It was primarily the close contact with adults who were engaged in them that was important. Through this, ASD managed to motivate them and gave them faith in themselves, that is, they believed that they were able to learn. ASD gave them coping experiences, and this may also have influenced positively on their self-esteem. The adolescents in this study have not felt that they have been recognised and valued at school; on the contrary, they have to a great extent felt like losers. They have had a problematic relationship with school for many years. The school years seem to have been a project without meaning. The majority of the pupils reported that school was boring, most of them had a bad relationship with the teachers and all of them had considerable difficulties with theoretical subjects. During the school year, five pupils left ordinary school; only four were still in ordinary schools at the end. Tom and Adrian, who moved to special schools, were incredibly satisfied with this. Finally, they felt that they did something meaningful. For Lars, who had begun working, life became much easier. The findings of this study may indicate that there exists both domination and resistance in schools. In accordance with Bourdieu, the school represents middle-class values, and pupils from this class will be more valued by their teachers than pupils who are less rich in cultural capital. Socially, the pupils coped ‘relatively well’, but this is not satisfactory. Because the credibility and ideals are middle-class founded, pupils with another habitus will constantly have an uphill struggle. The school is foreign for them; it is their ‘away ground’. The school as a middle-class arena has, and will still have, great problems with offering pupils in risk zones fundamental support. However, the findings may also indicate that pupils are intentional actors who demonstrate against the system. In some cases, this resulted in shirking school for a long period; being only physically present; and not paying any attention to the lessons. Others kicked up a row in class.

The principle ‘equality for all’ that ideally should exist in Norwegian schools seems to function inadequately. Ogden (1998, p. 7) claims that ‘the pupil's failure also is the school's failure’, when it cannot offer a qualitative, good and adjusted education. The public eye should therefore be directed towards the ordinary school as a problem in such a way that the complicated relationship between ‘the ordinary’ and ‘the special’ can be challenged.
